# Late development of coronary artery abnormalities could be associated with persistence of non-fever symptoms in Kawasaki disease

**DOI:** 10.1186/1546-0096-11-28

**Published:** 2013-07-31

**Authors:** Sayaka Fukuda, Shuichi Ito, Shinji Oana, Hirokazu Sakai, Hitoshi Kato, Jun Abe, Ryuko Ito, Akihiko Saitoh, John Ichiro Takayama

**Affiliations:** 1 Department of General Pediatrics and Interdisciplinary Medicine, National Center for Child Health and Development, 2-10-1 Okura, Setagaya-ku, Tokyo 157-8535, Japan; 2 Division of Nephrology and Rheumatology, National Center for Child Health and Development, 2-10-1 Okura, Setagaya-ku, Tokyo, Japan; 3 Division of Cardiology, National Center for Child Health and Development, 2-10-1 Okura, Setagaya-ku, Tokyo, Japan; 4 Department of Allergy and Immunology, National Center for Child Health and Development, 2-10-1 Okura, Setagaya-ku, Tokyo, Japan; 5 Department of Health Policy, National Center for Child Health and Development, 2-10-1 Okura, Setagaya-ku, Tokyo, Japan; 6 Division of Infectious Disease, National Research Center for Child Health and Development, National Center for Child Health and Development, 2-10-1 Okura, Setagaya-ku, Tokyo, Japan; 7 Department of Pediatrics, Niigata University Graduate School of Medical and Dental Sciences, 1-757 Asahi-machi, Chuo-ku, Niigata, Japan; 8 Department of Pediatrics, University of California San Francisco, 400 Parnassus Avenue, San Francisco, CA, USA

**Keywords:** Kawasaki disease, Coronary artery abnormalities, Fever, Intravenous immunoglobulin

## Abstract

**Background:**

Persistent fever after intravenous immunoglobulin (IVIG) is considered to be a major criterion of IVIG resistance in Kawasaki disease (KD), and a risk factor for the development of coronary artery abnormalities (CAA). However, the importance of persistent non-fever symptoms after defervescence has not yet been investigated. We examined the relationship between persistent non-fever symptoms and CAA in KD.

**Methods:**

We conducted a retrospective cohort study of patients hospitalized with KD at the National Center for Child Health and Development between 1 April 2008 and 31 March 2009. Patients were divided into two groups; group A included patients who still had non-fever symptoms one month after onset of the illness and group B included patients who did not have persistent non-fever symptoms. Demographic, clinical variables were compared between the groups.

**Results:**

Seventy-seven KD patients treated with IVIG were retrospectively analyzed. Patients were divided into two groups; group A included 12 (15.6%) patients and group B 65 (84.4%) patients. Demographic data, baseline laboratory data, and fever duration did not differ between the groups. In group A patients the most common persistent non-fever symptoms were lip erythema (n = 6) and bulbar conjunctivitis (n = 8). One month after onset of the illness CAA developed in seven of 77 patients (9.1%), four (33%) in group A and three (4.6%) in group B (odds ratio 10.3; 95% CI 1.9-54.8). Three patients in group A and one patient in group B developed CAA after the resolution of fever.

**Conclusions:**

Persistence of non-fever symptoms after IVIG may suggest persistence of latent inflammation, which may increase the risk of CAA. Therefore, patients with persistent non-fever symptoms may be at risk of developing CAA, even after defervescence. A prospective trial of additional IVIG for such patients should be considered.

## Background

Kawasaki disease (KD) was first described by Dr. Tomisaku Kawasaki in 1967 [1]. While the etiology remains unknown, the annual incidence of KD in Japan increased by 17% during 2005–2008 and currently nearly 12,000 new cases are reported each year [2]. High-dose intravenous immunoglobulin (IVIG) is administered as a standard initial treatment [3-5], but 10–20% of patients do not respond to IVIG. Because the effectiveness of the treatment of KD has been defined by the resolution of fever [6], IVIG resistance have focused more on the persistence of fever than on other KD symptoms [7-9]. Little is known about the impact of the persistence of non-fever symptoms on the development of coronary artery abnormalities (CAA). However, we have frequently observed patients whose non-fever KD symptoms remained after fever subsided. Most of them were not been received additional treatments, and some developed CAA during the subacute phase. The objective of our study was to determine the relationship between the persistent non-fever symptoms of KD and CAA.

## Methods

We conducted a retrospective cohort study of patients hospitalized with KD at the National Center for Child Health and Development between 1 April 2008 and 31 March 2009. The diagnosis of KD was based on the Diagnostic Guidelines for Kawasaki Disease [10]. We excluded patients whose symptoms resolved without IVIG. The remaining patients were evaluated one month after the onset of illness and were followed up for more than one year.

The following variables were obtained for each subject from the electronic medical records: sex, age, the numbers of major symptoms of KD, fever duration, and duration of hospitalization. Laboratory data collected prior to initial treatment included white blood cell count, absolute neutrophil count, hematocrit, platelet count, erythrocyte sedimentation rate, albumin, sodium, C-reactive protein (CRP), total bilirubin, aspartate aminotransferase, alanine aminotransferase, fibrinogen, and D-dimer.

The initial treatment for KD consists of aspirin 50 mg/kg/day, and IVIG 2 g/kg. For patients with persistent fever, additional treatments, including a second administration of IVIG, infliximab, and plasma exchange were considered.

Patients were classified into two groups based on the presence (group A) or absence (group B) of persistent non-fever symptoms at one month after the onset of illness. Fever was defined as axillary temperature >38°C measured using a digital thermometer. Presence of non-fever symptoms was defined the routine follow-up visit after hospital discharge: bilateral conjunctivitis, polymorphous exanthema, strawberry tongue or lip erythema, non-purulent cervical lymphadenopathy, and changes in the extremities. All subjects were examined in an outpatient clinic one month after the onset of illness and CAA were diagnosed using ultrasound based on the Japanese Ministry of Health, Labor and Welfare criteria [11].

Mann-Whitney test was used to determine the significance of demographic and clinical variables between the two groups. Differences were considered significant at the *p* < 0.05 level. The risk of developing CAA was determined using the chi-square test. All analyses were carried out using SPSS Statistics 17.0 (SPSS Japan, Tokyo). Approval of the study protocol was obtained from the Review Board of the National Center for Child Health and Development and written informed consent was obtained from the parents of the patients.

## Results

During the study period, 81 patients were diagnosed with KD. After excluding four patients whose symptoms resolved without IVIG, 77 patients were analyzed. Based on the persistence of non-fever symptoms, 12 patients (15.6%) were assigned to group A, and 65 patients (84.4%) were assigned to group B.

Persistent non-fever symptoms in group A patients included lip erythema (4/12), bulbar conjunctivitis (7/12), and both lip erythema and bulbar conjunctivitis (1/12). The groups did not differ based on sex, age, hospitalization date, the number of KD symptoms before the initial IVIG, fever duration, or duration of hospitalization (Table 1). Laboratory data obtained prior to the initial IVIG did not differ between the two groups (Table 2).

**Table 1:** Demographic and clinical variables

	**Group A (n = 12)**	**Group B (n = 65)**	***p*-value**
	**Median (Min–Max)**	**Median (Min–Max)**	
Sex (Male/Female)	M 7/F 5	M 42/F 23	N.S.
Age (months)	27 (4–65)	21 (2–9)	N.S.
Presenting symptoms (in KD criteria)	5 (5–6)	5 (3–6)	N.S.
Fever duration (days)	7 (4–10)	6 (4–15)	N.S.
Duration of hospitalization (days)	11 (7–38)	9 (6–25)	N.S.
CA involvement (n)	4	6	0.001*
Treatment			
Aspirin/Flurbiprofen (day of administration)†	4 (3–8)	5 (2–9)	N.S.
Initial IVIG (day of administration)†	4 (4–8)	5 (3–8)	N.S.
Second IVIG (n)	6	14	N.S.
Alternative Treatments	3rd IVIG	PE (2) Infliximab (1) Steroids (2)	
Total IVIG dosage (g/kg)	3 (2–6)	2 (2–4)	0.020**

Note: *Mann-Whitney test **chi-square test, odds ratio 10.3 (95% CI 1.9–54.8).

†Day calculated by subtrating date fever began from date treatment began.

*PE* Plasma Exchange, *N.S*. Not significant.

**Table 2:** Baseline laboratory tests (prior to treatment)

**Variables**		**Group A**	**Group B**	**p-value**
**Median (Min–Max)**				
WBC	(/μL)	12190.0 (6760.0–20640.0)	12890.0 (5080.0–30160.0)	N.S.
Neutrophil	(/μL)	7891.2 (4191.2–13130.1)	8659.0 (3518.1–26771.2)	N.S.
Hematocrit	(%)	33.2 (27.4–37.9)	31.7 (25.1–41.1)	N.S.
Platelet	(×104/μL)	36.3 (2.37–54.7)	38.6 (20.0–86.9)	N.S.
Total Bilirubin	(mg/dL)	0.7 (0.3–2.4)	0.6 (0.1–4.6)	N.S.
AST	(IU/L)	93.0 (22.0–290.0)	42.0 (20.0–648.0)	N.S.
ALT	(IU/L)	184.5 (14.0–364.0)	42.0 (7.0–606.0)	N.S.
Sodium	(mEq/L)	135.0 (130.0–139.0)	136.0 (130.0–143.0)	N.S.
CRP	(mg/dL)	8.8 (4.2–15.3)	7.4 (0.8–18.7)	N.S.
Albumin	(g/dL)	3.5 (2.8–3.8)	3.5 (2.5–4.3)	N.S.
D-dimer	(μg/mL)	1.9 (1.0–8.9)	1.3 (0.5–10.3)	N.S.
Fibrinogen	(mg/dL)	379.0 (550.0–875.0)	607.0 (392.0–901.0)	N.S.
ESR (60 min)	(mm)	100.5 (53.0–130.0)	99.0 (38.0–140.0)	N.S.

Note: Mann-Whitney test, N.S.: not significant.

*WBC* White blood cell, *AST* Aspartate aminotransferase, *ALT* Alanine aminotransferase.

*CRP* C-reactive protein, *ESR* Erythrocyte sedimentation rate.

With regard to treatment, the initiation date of IVIG and aspirin administration did not differ, but the total IVIG dose was higher in group A compared with group B (*p* = 0.02). A second course of IVIG was administered to six of the 12 patients (50%) in group A and to 14 of the 65 patients (22%) in group B. One patient in group A received a third dose of IVIG, and in group B two patients received prednisolone, one patient received infliximab, and two patients received plasma exchange (Table 1).

Seven of 77 patients (9.1%) developed CAA, four of the 12 patients (33.3%) in group A and three of the 65 patients (4.6%) in group B (Table 3). Among them, three patients in group A and one patient in group B developed CAA after their fever subsided. Of particular interest, one patient in group A suffered from progressively worsening CAA that resulted in a giant coronary aneurism during afebrile convalescence. Two other patients in group A had elevated CRP levels one month after the onset of illness. The adjusted odds ratio for CAA in group A patients was 10.3 (95% CI 1.9–54.8, *p* = 0.001) compared with patients in group B. With regard to the long term prognosis of CAA in these patients, at one year after the onset of KD, two patients in group A still had coronary artery dilatation or aneurysm, and three patients in group A and two patients in group B had been treated with anticoagulant therapy.

**Table 3:** Details of patients who developed coronary artery abnormalities

** Case no.**		**Sex**	**Onset age (months)**	**Symptom at 1 month after onset**	**Maximum size of CA lesion (mm)**							**CRP at 1 month after onset (mg/dl)**	**Anticoaglant therapy at 1 year after onset**
					**At defervescence**			**At 1 month after onset**		**At 1 year after onset**			
					**Day**	**RCA**	**LCA**	**RCA**	**LCA**	**RCA**	**LCA**		
Group A	1	F	4	conjunctivitis lip erythema	13	1.7	2.0	3.3	2.1	2.1	1.9	4.0	Yes
	2	F	11	lip erythema	10	2.2	1.8	4.5	4.5	3.0	2.3	2.9	Yes
	3	M	47	conjunctivitis	12	2.6	2.5	4.5	8.7	2.5	2.5	≤0.2	Yes
	4	M	17	lip erythema	12	3.4	7.5	2.8	7.8	2.7	4.5	n.d.	No
Group B	5	M	28	None	18	3.1	3.5	2.6	3.4	2.0	2.2	n.d	Yes
	6	M	37	None	9	2.0	2.8	2.5	3.3	2.6	2.9	≤0.2	No
	7	M	4	None	7	2.1	1.9	4.2	4.4	2.2	2.2	n.d.	Yes

Note: *CA* Coronary artery, *LCA* Left coronary artery, *RCA* Right coronary artery, *n.d.* Not done.

## Discussion

Although the efficacy of IVIG in reducing the incidence of CAA in KD is well documented, 10–20% of patients do not respond to the initial administration of IVIG. Many researchers have attempted to identify risk factors associated with IVIG resistance based on the persistence of fever, but not on the other signs and symptoms of KD [12-14]. However, in practice, we have managed patients who had lip erythema and/or bulbar conjunctivitis long after their fever had subsided. In our retrospective study of 77 patients 57 attained defervescence after their first IVIG, but of these 57 patients 12 (15.6%) had one or two of the non-fever symptoms of KD at their follow up visit a month after the onset of illness. Importantly, four of these 12 patients (33.3%) developed CAA. Of these four patients, three developed CAA after defervescence had been attained. Of note, CRP levels in two of these patients remained elevated at one month after the onset of illness, whereas all the patients in group A who did not develop CAA had CRP values less than 0.2 mg/dl (data not shown). Although one patient in group B also developed CAA, our findings suggest that defervescence alone is not sufficient to define the responsiveness of KD patients to IVIG. If patients show persistent non-fever symptoms after defervescence, cardiac ultrasound and inflammatory laboratory markers should be closely monitored. The adjusted odds ratio of developing CAA in this group of patients was as high as 10.3 compared with the patients who attained defervescence with no persistent non-fever symptoms after initial IVIG (*p* = 0.001).

At present, we do not understand the pathogenesis of the prolonged non-fever symptoms. The initial day of IVIG treatment and the baseline laboratory data were not significantly different between the two groups. The patients in group A received a higher total dosage of IVIG than patients in group B, but the additional treatment was primarily directed against fever. The duration of fever was not different between the two groups. While the resolution of fever after IVIG may indicate improvement in systemic inflammation, the persistence of non-fever symptoms may suggest the presence of localized vascular inflammation, including in the coronary arteries, even after patients have achieved defervescence. The elevated CRP observed in some patients at one month after the onset of illness supports this hypothesis. Therefore, we believe that the definition of IVIG resistance should be reconsidered to include the persistence of non-fever symptoms as well as fever.

Our study has several limitations. First, we could not observe the patients regularly after their hospital discharge and the presence or absence of non-fever symptoms was primarily determined by one of the attending physicians at an outpatient clinic. However, persistence of non-fever symptoms in patients who developed CAA was evaluated by at least two physicians. Second, we did not treat patients who did not respond to initial IVIG using a standardized protocol. As discussed in the Results section, the patients who did not achieve defervescence after the second IVIG were given additional treatment. The different additional modalities may have affected the incidence of CAA in these patients. However, more refractory patients might have been included in group B, because five patients were treated with prednisolone (n = 2), infliximab (n = 1) and plasma exchange (n = 1). Third, we examined only a small number of patients admitted to one institution in this study. Therefore, this small cohort study still has limitation to conclude that persistent non-fever symptoms should be included as one of the definitions of IVIG resistance, and such patients may be at risk of developing CAA, even after defervescence.

## Conclusion

Our study demonstrated that among patients who attained defervescence after their first IVIG, a certain percentage had one or two of the non-fever symptoms of KD a month after the onset of illness. The persistence of these non-fever symptoms may be an important risk factor for CAA and may indicate the need for further treatment. Patients with persistent non-fever symptoms after defervescence require close and continuous follow-up of cardiac ultrasound and inflammatory laboratory markers. However, prospective cohort study with larger number of patients could be necessary to confirm our result.

## Abbreviations

KD: Kawasaki disease; IVIG: Intravenous immunoglobulin; CAA: Coronary artery abnormalities.

## Competing interests

The authors declare that they have no competing interests.

## Authors’ contributions

SF wrote the first draft. SF, SO, SI, HK and AS saw the patients that took part in this study. HS is a division director of general pediatrics and adviser of this study. JA developed the study design, RI is a statistician and adviser of statistics and JIT is the overall supervisor of this study. All authors read and approved the final manuscript.
